# Priced out of belonging? Insufficient concessions on membership fees across international societies in ecology and evolution

**DOI:** 10.1098/rspb.2024.1430

**Published:** 2025-02-05

**Authors:** Malgorzata Lagisz, Kevin R. Bairos-Novak, April Robin Martinig, Michael G. Bertram, Ayumi Mizuno, Saeed Shafiei Sabet, Matthieu Paquet, Manuela S. Santana, Eli S. J. Thoré, Nina Trubanová, Joanna Rutkowska, James A. Orr, Elina Takola, Yefeng Yang, Patrice Pottier, Dylan G. E. Gomes, Ying-Chi Chan, Zhenzhuo Xian, Caleb Onoja Akogwu, Szymon M. Drobniak, Shinichi Nakagawa

**Affiliations:** ^1^ School of Biological, Earth and Environmental Sciences, University of New South Wales, Sydney, NSW 2052, Australia; ^2^ Department of Biological Sciences, University of Alberta, Edmonton, Alberta, Canada T6G 2E9; ^3^ School of the Environment, University of Queensland, Brisbane, QLD 4072, Australia; ^4^ Department of Biology, University of British Columbia, 1177 Research Road, Kelowna, British Columbia, Canada V1V 1V7; ^5^ Department of Wildlife, Fish, and Environmental Studies, Swedish University of Agricultural Sciences, Umeå 907 36, Sweden; ^6^ Department of Zoology, Stockholm University, Stockholm, Sweden; ^7^ School of Biological Sciences, Monash University, Melbourne, Australia; ^8^ Fisheries Department, Faculty of Natural Resources, University of Guilan, PO Box 1144, Sowmeh Sara, Iran; ^9^ CNRS Theoretical and Experimental Ecology Station (SETE), UAR 2029 2 route du CNRS, Moulis 09200, France; ^10^ Centre for Marine Studies, Federal University of Paraná, Pontal do Paraná, State of Paraná 83255-000, Brazil; ^11^ Laboratory of Adaptive Biodynamics, Research Unit in Environmental and Evolutionary Biology, Institute of Life, Earth, and Environment, University of Namur, Rue de Bruxelles 61, Namur 5000, Belgium; ^12^ TRANSfarm—Science, Engineering, & Technology Group, KU Leuven, Lovenjoel 3360, Belgium; ^13^ School of Biology and Environmental Science, University College Dublin, Belfield, Dublin 4, Ireland; ^14^ Faculty of Biology, Institute of Environmental Sciences, Jagiellonian University, Gronostajowa 7, Kraków 30-387, Poland; ^15^ Department of Biology, University of Oxford, Oxford, UK; ^16^ Department of Computational Landscape Ecology, Helmholtz-Centre for Environmental Research-UFZ, Permoserstraße 15, Leipzig 04318, Germany; ^17^ Division of Ecology and Evolution, Research School of Biology, The Australian National University, Canberra, ACT 2600, Australia; ^18^ Ocean Ecology Lab, Marine Mammal Institute, Oregon State University, Newport, OR 97365, USA; ^19^ Swiss Ornithological Institute, Seerose 1, Sempach 6204, Switzerland; ^20^ University of Chinese Academy of Sciences, Beijing 100049, People's Republic of China; ^21^ CAS Key Laboratory of Plant Germplasm Enhancement and Specialty Agriculture, Wuhan Botanical Garden, Chinese Academy of Sciences, Wuhan 430074, People’s Republic of China

**Keywords:** career barriers, equity, diversity and inclusion, meta-research, open science, professional and academic organizations

## Abstract

Learned societies, as professional bodies for scientists, are an integral part of the scientific system. However, their membership fees have the potential to be prohibitive to the most vulnerable members of the scientific community. To shed light on how membership fees are structured, we conducted a survey of 182 international learned societies relevant to researchers in ecology and evolution. We found that 83% of these societies offered fee concessions to students, but only 26% to postdoctoral researchers. An average regular membership fee—US$67.8, student fee—US$27.4 (42.7% of the regular fee) and postdoctoral fee—US$42.7 (52.9%). Other types of individual concessions, such as for emeritus, family or unemployed, were rare (2–20%). Of the surveyed societies, 43% had discounts for members from developing countries (Global South). Such discounts were more common among societies located in high-income countries. Societies with a publicly visible commitment to equity, diversity and inclusion were more likely to offer different types of concessions. Currently, fees may prevent researchers from vulnerable and underprivileged groups from accessing multiple professional benefits offered by learned societies in ecology and evolution. This includes postdoctoral researchers, who should receive more support. We recommend tangible actions towards making learned societies more affordable and accessible.

## 1. Introduction

The need to belong is fundamental to humans. Being part of one or multiple groups is not just a matter of personal self-worth [1] but is beneficial for social and professional success. Filling this space, professional organizations for scholars and academics of various kinds (herein referred to as ‘learned societies’) have existed for several hundred years—for example, the Royal Society of London for Improving Natural Knowledge dates back to the mid-seventeenth century. From the very start, learned societies brought together like-minded people, fostering scientific communication, gradually expanding organizations’ core missions and functions, branching into specialized areas, but also merging and growing into powerful institutions and communities [2,3]. What unites various learned societies, from local to international, and from specialist to interdisciplinary, is a dedication to innovation and/or community support, as often proclaimed in their mission and vision statements [4].

The last several decades have seen a growing commitment to address inequities and biases omnipresent in science and academia [5,6]. In this regard, equity, diversity and inclusion (EDI) committees and officers are expected to initiate and oversee policies and actions that aim to recruit and support members from historically and currently underrepresented and underserved groups and backgrounds in science [7]. We are beginning to see more dedicated awards and prizes, networking events, mentoring programmes, travel grants, discounts for attending meetings, workshops, and courses targeted towards marginalized and underprivileged groups [8].

Before gaining the support and opportunities provided by learned societies, one usually needs to become a member by paying membership fees. Fees exist to support societies and their activities, such as meetings, grants, awards and general administrative costs. However, membership fees can be a barrier for some, preventing them from joining or renewing their membership [9]. Considering this, societies can introduce concessions, usually in the form of fee discounts or waivers. Such concessions can target groups of members that have traditionally been perceived as being the least likely to afford membership fees—for example, students, early-career (postdoctoral) researchers or retirees. Furthermore, societies with an international membership base can differentiate their fees based on the country of residence of prospective members. Other types of potential concessions can be based on personal circumstances, such as current income levels.

Practices related to making membership accessible differ among societies [10]. However, when societies fail to consider multiple factors that can influence the affordability of fees, they may propagate and reinforce existing group-level and individual biases in academia. Specifically, by making membership financially inaccessible, societies could contribute to the ‘Matthew effect’ where relatively privileged groups become more privileged by gaining access to more resources and opportunities through cumulative advantage [11]. Conversely, people with limited financial resources and who are not eligible for special considerations to apply for society memberships could miss out on career-building opportunities, advice, inspiration, networking and community and can slide further behind through cumulative disadvantage.

Societies can change their fee structures to improve accessibility. Such actions are likely aligned with greater awareness and commitment to fostering EDI in science [5]. Recognizing EDI as central to membership affordability could trigger a cascade of positive change, where an increasing number of societies would implement more inclusive membership practices, especially if they can see that such practices have already been successfully implemented by highly respected organizations [12].

Here, we focus on the fields of ecology and evolution to examine EDI questions in membership fee structures. Ecology and evolution both are hyper-diverse fields, drawing researchers from various countries and institutions from around the globe to international learned societies. However, it is likely that most societies originate or exist to date as elite institutions in more developed countries (represented by the Global North), which can affect their accessibility for members from other regions and underrepresented groups. Thus, a thorough evaluation of the range and inclusiveness of fees charged by ecology and evolution societies is warranted.

## 2. Aims and approach

The overarching aim of this work is to collate relevant evidence and advocate for change. To achieve this aim, we conducted a survey focused on current practices related to structuring membership fees across international learned societies that are broadly relevant and/or popular among ecology and evolutionary biology researchers. From publicly available information, we collected data on membership fee structures and amounts, as well as auxiliary data on the learned societies themselves, and advertised membership benefits. We used this data to answer the following research questions, grouped into five themes:

1. *Individual full membership, student and postdoctoral researcher fees*: What are currently the standard individual membership fees, student membership fees and postdoctoral/early-career researcher membership fees? By how much are the concession fees reduced relative to the standard membership fee? For how many years are postdoctoral researchers eligible for discounts (in terms of the number of years post-PhD or the total number of eligibility years)?
2. *Country-level fee discounts and waivers*: What is the geographical distribution of the locations (headquarters/registration/incorporation country and continent) of the international societies in ecology and evolution? Does geographical distribution affect membership pricing? Are discounted or waived fees available for individual members from some countries or regions? How are such countries or regions defined? Do societies from countries with developed economies discount rates for members from countries with developing economies? Do societies from countries with developing economies increase their rates for members from other countries?
3. *Individual-level discounts and waivers*: Are discounted individual membership fees currently available for the following groups: students, postdoctoral researchers, retired/emeritus, unemployed, employed part-time, junior (pre-university), family, educators/outreach/communication non-academic specialists, general community/public or any other groups? Are complete or partial individual membership fee waivers currently available on individual request? How are they defined and who is eligible?
4. *Societies’ EDI characteristics*: Do societies with a commitment to EDI state on their website or in their policy documents (or having dedicated EDI structures) offer a more inclusive individual membership fee structure (e.g. lower fees, more options for concessions)?
5. *Membership benefits*: What are the tangible benefits of individual society membership (e.g. opportunities to apply for awards, travel grants, conference fee discounts, journal subscription discounts)?

## 3. Methods

This project is registered with the Open Science Framework (https://osf.io/r3764/?view_only=a7d7b54cfd434ca69a26c58f0f0281c9). We developed the protocol during a Society for Open, Reliable, and Transparent Ecology and Evolutionary Biology (SORTEE) hackathon, which was held online on 18 October 2023. In electronic supplementary material, table S1, we present working definitions of the key terms used to define the scope of our work and inclusion criteria for data collection. Note that we consider a society to be ‘international’ if it has international reach, including having (or claiming to have) international chapters or activities in collaboration with societies from other countries. Additional methodological details are provided in the electronic supplementary material, supplementary information files.

### (a) Data compilation

We conducted a survey focused on current practices related to membership fees across international learned societies related to ecology and evolution (including whole-organism biology and ecosystem/environmental sciences). To compile the initial long list of potentially relevant societies, we consulted related literature [13], checked societies associated with journals from the SCImago category ‘Ecology, Evolution, Behaviour, and Systematics’, searched societies listed on Wikipedia and received specialists’ recommendations. This resulted in a long list of 215 societies, which is provided in the registered OSF protocol (https://osf.io/r3764/?view_only=a7d7b54cfd434ca69a26c58f0f0281c9). We then excluded societies without the option of individual membership (e.g. societies that are aggregations of other societies or only offer institutional memberships), inactive societies and societies without any international aspects or activities, as judged from publicly available online documents. After this initial screening, 184 societies remained for data extraction. During data extraction, after further examination, another two societies were deemed ineligible (one due to not being relevant to ecology or evolutionary biology and one as not being international).

### (b) Data collection items

Electronic supplementary material, table S2, presents a detailed list and descriptions of extracted data items. In brief, the extracted items included society identity information (full name, webpage address, country of its headquarters/registration/incorporation), scope of its activities/membership (society type) and presence of EDI statements or structures on the society website. We then extracted data on each society’s individual membership fee structure: the amount of annual regular fee, postdoctoral researcher and student fees, fee currency, types of other discounted membership fees available (namely: retired/emeritus, unemployed, family, junior, community, professional, other) and other characteristics of the fee structure, including currency. We also coded six categories of advertised society membership benefits (namely: conference registration discount or waiver; funding (e.g. travel awards/grants, research funding, prizes); journal subscription discount or waiver; publication fees (article processing charges (APC)) discount or waiver; networking or professional development (e.g. membership platform, mentoring, exclusive webinars, workshops, training courses); other). We accompanied coded data with comments on the context (e.g. Web links, text quotes) and notes on justifications and assumptions made when extracting data to make the data extraction process replicable. We extracted all data in duplicate (i.e. two individuals independently extracted data from each society) after an initial round of piloting and training on three randomly selected societies.

### (c) Data analysis

We analysed the final consensus dataset using R computational environment v. 4.3.2 [14] in RStudio v. 2023.12.0+369. Full session information, including R packages used and all R code and outputs, is included in electronic supplementary material, supplementary file 1.

During data processing, we first removed data on societies that were deemed ineligible at the data extraction stage. We then counted and removed data on nine societies that did not have any publicly available information on their fees, and four societies that offered free membership for anyone (and, thus, had no fee structure). For the remaining data, we converted all recorded fee values (for standard/regular/full individual membership, student membership and postdoctoral researcher membership) from their original currencies into United States dollars (USD). We used USD exchange rates from 23 February 2024, as listed on Google Finance (https://www.google.com/finance/).

We then followed the steps outlined in the registered protocol (https://osf.io/r3764/?view_only=a7d7b54cfd434ca69a26c58f0f0281c9) to summarize and visualize data across 169 societies to answer our pre-planned research questions. We summarized the dataset by extracted categorical variables and visualized pooled data relevant to each of our main questions. In brief, we examined the relationship between the full fee amount and the amounts charged for two main types of concession fees (student and postdoctoral researcher). We compared the fee amounts between societies based in Global North versus Global South countries, using the United Nations List of Global South Countries (https://worldpopulationreview.com/country-rankings/global-south-countries). Furthermore, we tested the association between the presence of EDI statements/structures and the amount of student and postdoctoral researcher discount relative to the full membership fee. Finally, we examined the association between the presence of EDI statements/structures and the numbers of discount categories, as well as the presence of country-level fee discounts, increases, and other kinds of concessions coded in our dataset.

### (d) Deviations from the protocol

We followed our study protocol with four exceptions and additions. First, during data extractions, we additionally coded which societies did not publicly present any information on their fees (e.g. fees or membership were mentioned, but fee descriptions were missing, claimed to be in preparation or temporarily suspended, membership is obtained by attending a conference/meeting, fees information only available upon request). Second, we coded which societies had their fees publicly shown in more than one currency. Third, instead of a Chi-square test, we used a Fisher’s exact test for count data, due to small sample sizes [15]. Fourth, when comparing the fees of societies with and without EDI statements, instead of logistic regression, we used two-sample *t*-tests for independent samples and without assuming equal variances. This is because we assume that the presence or absence of EDI statements is more likely to drive or be associated with differences in fees across learned societies rather than the other way around.

## 4. Results

Our dataset consists of 182 societies that fulfilled our inclusion criteria. However, nine societies did not present extractable information on their individual fee amounts (the Australasian Evolution Society, the Asian Society of Vector Ecology, the Gazi Entomological Research Society, the International Network for the Study of Asian Ants, the International Society for Systems Biology, the Iranian Society of Ichthyology, the Romanian Society of Palaeontologists, the Latin American Society of Bryology and the Society for Vector Ecology) and four societies offered free membership to everyone (the European Ornithologists’ Union, the European Pond Conservation Network, the International Association for Ecology and the International Council for the Exploration of the Sea), thus we could not extract the fee discounts data for these societies. For the remaining 169 societies, we present detailed results structured by the five themes of our project below.

### (a) Individual full membership, student, and postdoctoral researcher fees

**Figure 1.**
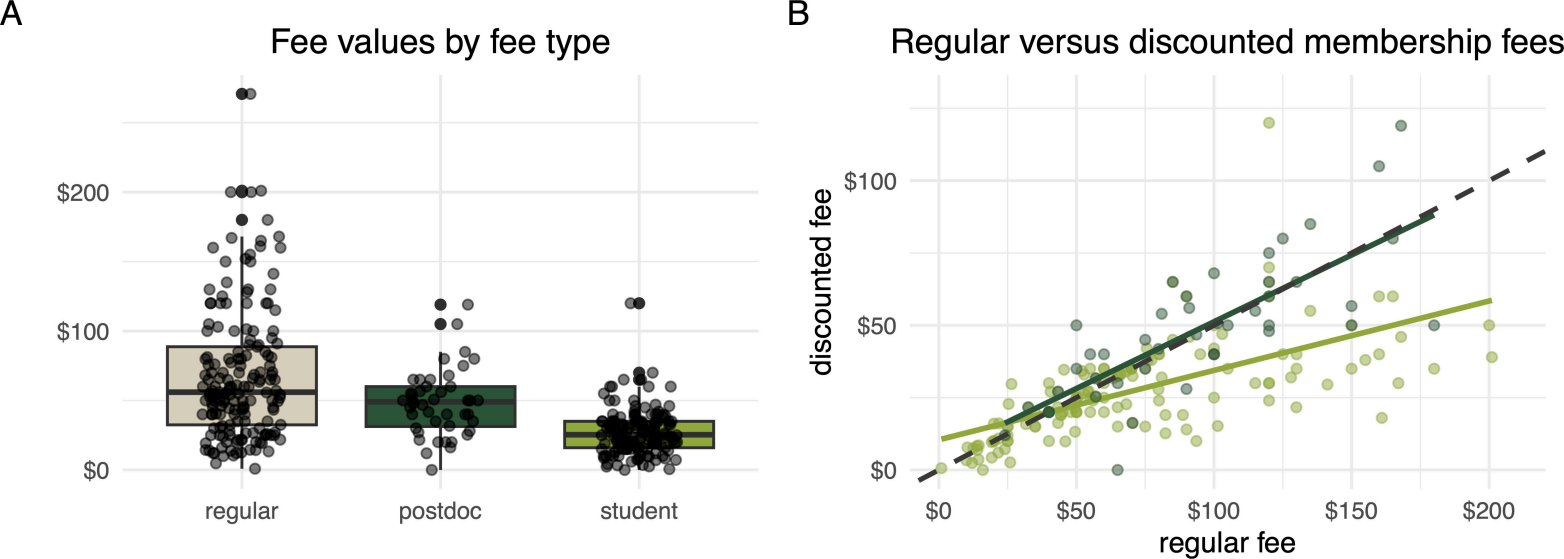
Comparisons of the three main categories of indvidual membership fees across 169 learned societies related to ecology and evolutionary biology. (A) Distribution of the monetary amounts (in USD) of regular, student and postdoctoral researcher individual membership fees. (B) Regular versus discounted fees (in USD) for students (lighter green) and postdoctoral researchers (darker green). The dashed diagonal line represents a 50% concession (discount) as a reference. ‘Postdoc’ stands for ‘postdoctoral researcher’.

Figure 1*a* shows data on fees charged in three individual membership categories across the 169 included societies with usable data (see above). Regular (full) individual membership ranged from 1 to 271 USD per year (mean = 67.8, median = 56.0). Student memberships were offered by 141 societies (83.4%) and ranged from 0 to 120 USD per year (mean = 27.4, median = 25.3). Student fees were typically around 40% of the regular fee (mean = 42.7, median = 44.4; figure 1*b*). Only 44 societies (26.0%) offered postdoctoral researcher memberships, which varied in price from 0 to 119 USD per year (mean = 48.0, median = 49.0), and were around 50% of the regular fee (mean = 52.9, median = 50; figure 1*b*). Out of 44 societies with postdoctoral researcher concessions, 15 reported eligibility timeframes for this member category, which were typically around 5 years (range = 3–8 years, mean = 4.9, median = 5 years post-PhD). Overall, fees higher than 50 USD were common for regular members (56.8% of the surveyed societies) and postdoctoral members (38.6%), but uncommon for student members (6.4%). On top of the membership fees, 55.0% of the surveyed societies accepted voluntary monetary donations, usually through a link from their website to a payment portal.

### (b) Country-level fee discounts and waivers

The 169 societies in our dataset were formally linked (e.g. incorporated/registered) to 28 countries across six continents. However, the USA (50%) followed by the UK (12%) were the two dominating countries (electronic supplementary material, figure S1). This was also reflected in the frequencies of the listed currencies of the membership fees (USD 54%, EUR 15% and GBP 11%; electronic supplementary material, figure S2). Six Global South countries (India, Argentina, Kenya, South Africa, Brazil, and The Philippines) were the base countries of 18 societies in our dataset (11%).

**Figure 2.**
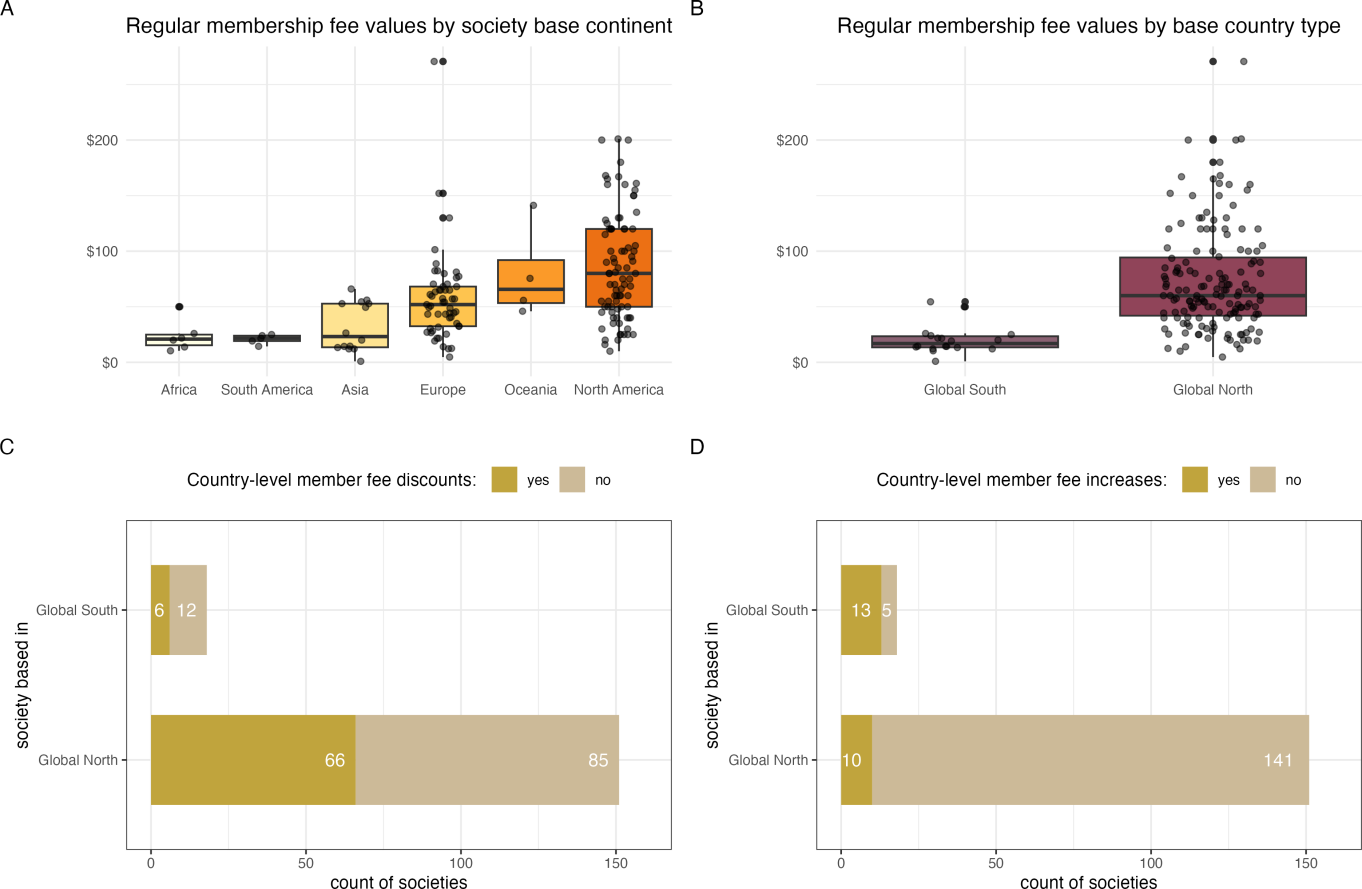
Society base country and membership fees across 169 learned societies related to ecology and evolutionary biology. (A) Amounts (in USD) of regular individual membership fees according to the continent on which each society is based. (B) Amounts (in USD) of regular individual membership fees according to whether the society is based in a Global South or Global North country. (C) Availability of discounted or waived fees for members from other countries according to whether the society is based in a Global South or Global North country. (D) Imposed increased fees for members from other countries according to whether the society is based in a Global South or Global North country.

Societies’ locations were linked to membership pricing. The average price (USD) of regular individual membership was lowest for societies based in Africa, South America and Asia (figure 2*a*). A similar pattern was evident when base countries were grouped into Global South and Global North categories (figure 2*b*), with Global North having significantly higher regular membership fees (Cohen’s *d* = 1.55, *n*_GN_ = 151, *n*_GS_ = 18, *t* = 10.8, *p* < 0.001).

Country-level concessions were common. Overall, 43% of societies offered discounted or waived fees for individual members residing in selected countries or regions (electronic supplementary material, figure S3). Such countries or regions were usually defined in the eligibility criteria using words related to the country’s economic development status or average personal income levels (electronic supplementary material, figure S4). Societies based in the Global North or Global South offered country-level membership concessions at similar rates (Fisher’s exact test for count data: OR = 0.65, 95% CI = 0.188–1.978, *p* = 0.458; figure 2*c*). Conversely, 13% of societies imposed higher than regular fees on individual members from other countries or regions (electronic supplementary material, figure S5). Such countries or regions were defined in the eligibility criteria using words related to the member’s country of residence being outside, overseas or foreign to the society’s base country (electronic supplementary material, figure S6). Societies based in countries classified as Global South more often imposed increased fees for members from outside their country or region than Global North societies (Fisher’s exact test for count data: OR = 34.823, 95% CI = 9.490–151.962, *p* < 0.001; figure 2*d*).

### (c) Individual-level discounts and waivers

**Figure 3.**
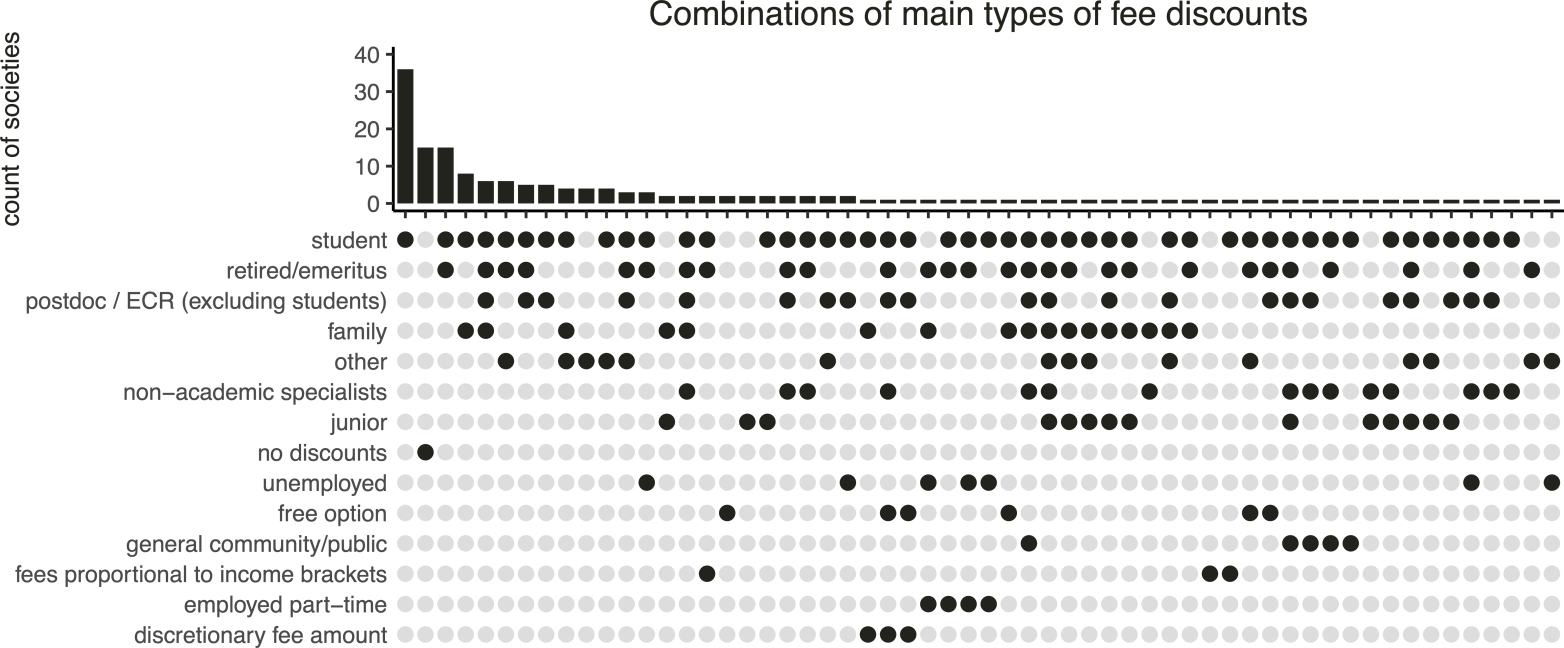
Combinations of the main types of individual-level concessions (discounts) across 169 learned societies related to ecology and evolutionary biology.

Societies varied in types and combinations of individual-level discounts (figure 3). Student discounts were, by far, the most common (82%; electronic supplementary material, figure S7). Retired and emeritus members came next, but were not ubiquitous (38%). Postdoctoral researchers could get fee discounts in only a quarter of societies (24%) and family members in a fifth (20%). The fee category coded as ‘other’ appeared in 19% of the societies, but it was a composite of diverse types of concession memberships, such as honorary, group, institutional, lifelong, multi-year, donation and some unclear options. Concession types that had limited representation supported non-academic specialists, young, unemployed, employed part-time and members of the general community/public (3−11%). Only four societies (3%) structured their fees using a ‘sliding scale’ approach with fees proportional to personal income brackets. However, seven societies (4%) had a ‘free’ option and three societies (2%) allowed members to pay however much they could afford (discretionary fee amounts). This contrasts to the approach taken by 15 societies (9%) that offered no discounts of any kind.

One-third (36%) of the societies offered only one type of discount (electronic supplementary material, figure S8), usually for students. One-quarter (25%) offered two and one-fifth (20%) offered three types of discounts. Societies that offered more than three types of discounts made up the remaining 19%. On top of this, 15% of societies offered complete or partial fee waivers on individual requests. However, such on-demand fee waivers were sometimes exclusive to students or residents of developing countries, racial or ethnic minorities, or were limited to a maximum duration of 1 year. They typically required a written application with justification for the waiver request. One society offered potential fee waivers in exchange for in-kind contributions.

### (d) Societies’ equity, diversity and inclusion characteristics

**Figure 4.**
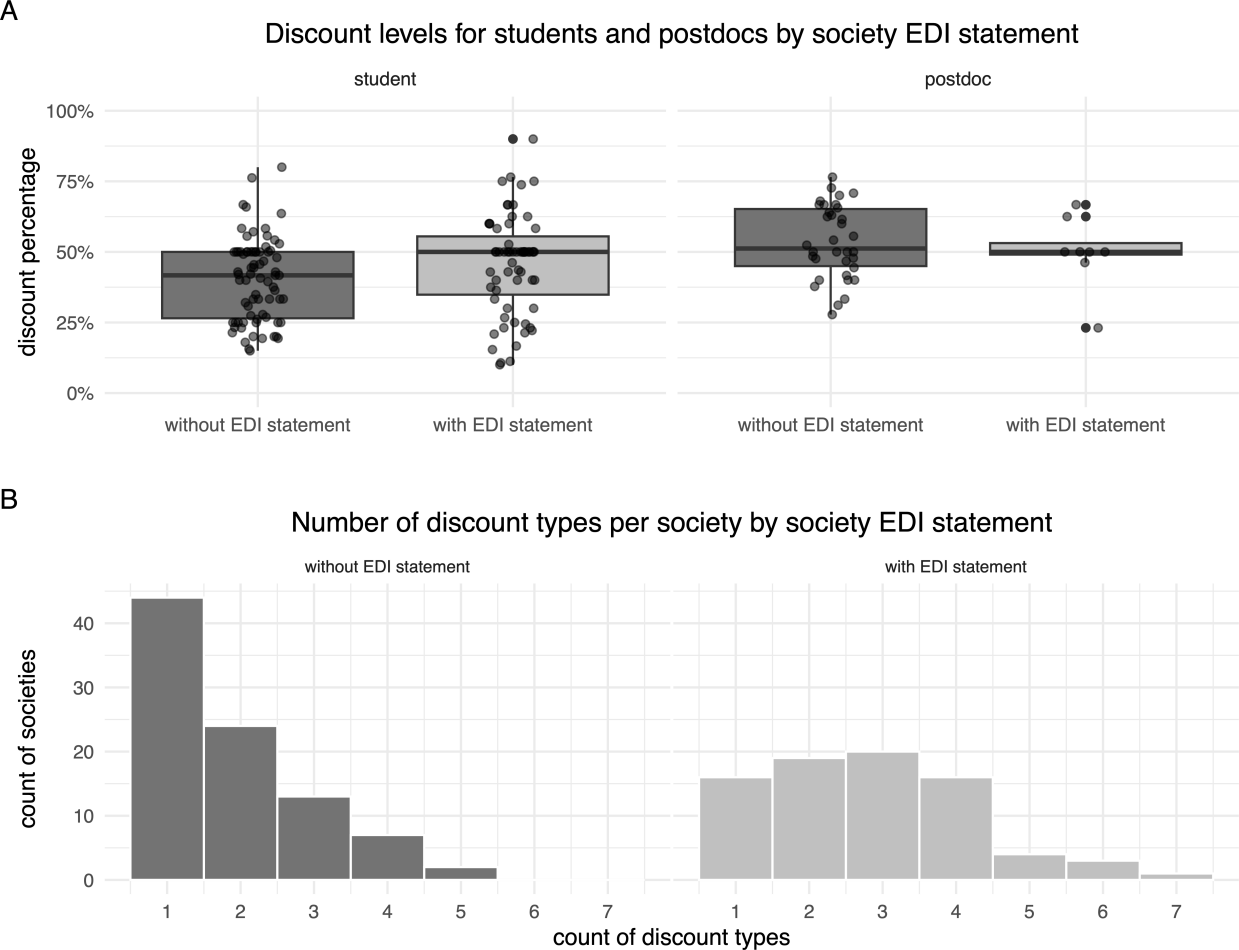
Membership fee concessions (discounts) in societies with and without public EDI structures across 169 learned societies related to ecology and evolutionary biology. (A) Student and postdoctoral researcher (postdoc) membership fees as a percentage of the regular membership fee. (B) Distributions of the number of available types of concessions (discounts) per society, as classified in our survey.

Around half (47%) of the societies publicly expressed their commitment to EDI on their website or policy documents. These societies usually also had EDI-dedicated structures, such as a committee or officers (37%; Fisher’s exact test for count data: OR = 133.0, 95% CI = 30.88–1198.52, *p* < 0.001; electronic supplementary material, figure S10; due to this strong overlap, we focused on EDI structures only thereafter) and were more likely to be based in Global North countries (41% of societies from Global North versus 0% of Global South: OR < 0.01, 95% CI = 0.00–0.34, *p* < 0.001; electronic supplementary material, figure S11). On average, societies with and without public EDI structures had similar relative levels of student and postdoctoral researcher concessions (figure 4*a*). In contrast, societies with EDI structures often had membership fees with more options for discounts in comparison to societies without EDI structures (figure 4*b*). The former were also more likely to offer country-level discounts (Fisher’s exact test for count data: OR = 4.74, 95% CI = 2.32–9.92, *p* < 0.001; electronic supplementary material, figure S12), and less likely to impose increased fees for members from outside their country or region (Fisher’s exact test for count data: OR = 0.14, 95% CI = 0.02–0.60, *p* = 0.002; electronic supplementary material, figure S13). Furthermore, EDI structures were associated with a higher presence of fee waivers on individual request, and concessions for students, postdoctoral researchers, retirees and non-academic specialists (electronic supplementary material, figures S18). In contrast, we found no association with discounts for part-time or unemployed researchers, general community/public, family, junior, sliding scale, discretionary fee amounts, or a ‘no fee’ option (electronic supplementary material, figures S27).

### (e) Membership benefits

**Figure 5.**
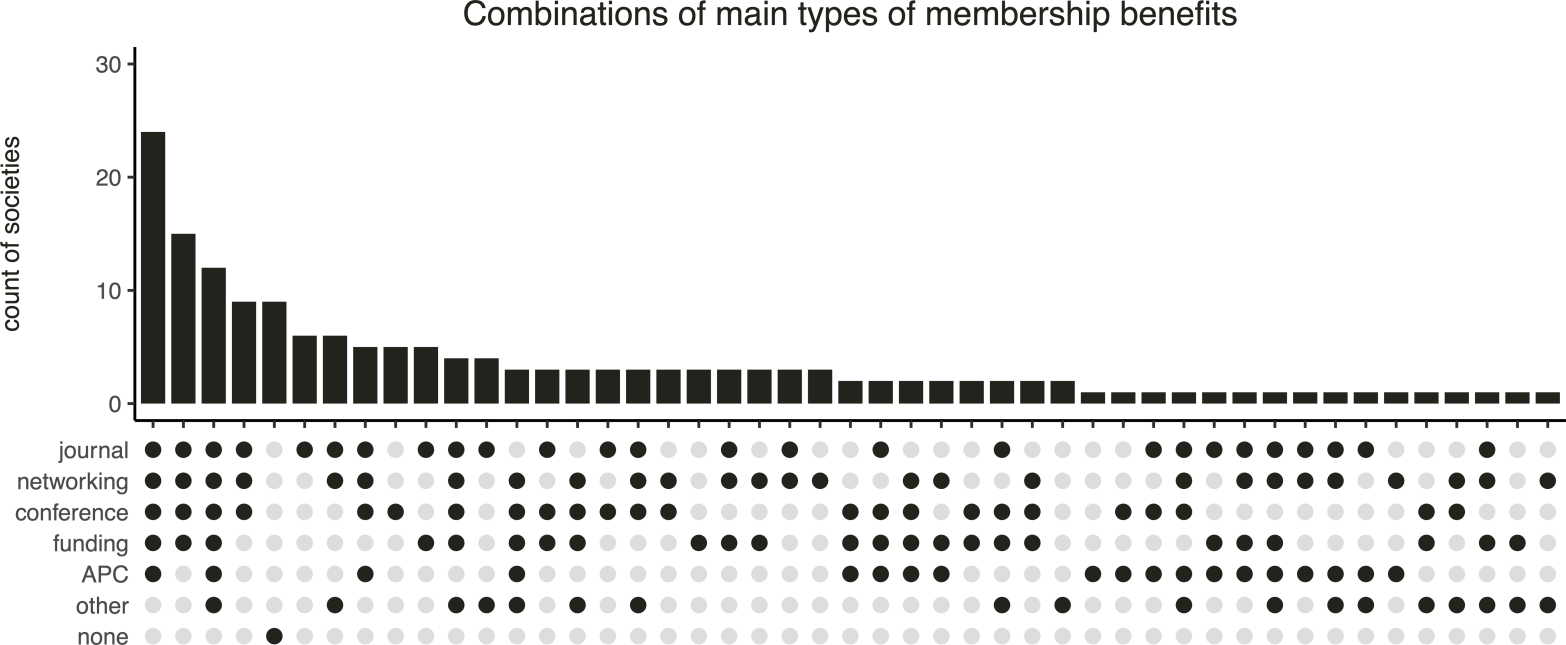
Combinations of the main types of individual membership benefits across 169 learned societies related to ecology and evolutionary biology. ‘None’ stands for societies that did not publicly describe any benefits for their members.

Almost all (95%) of the included societies publicly listed tangible benefits provided for their members. These benefits were grouped into six categories during data extraction. Among the six categories, free or discounted journal subscriptions were the most common (70%; electronic supplementary material, figure S35), followed closely by various networking opportunities (67%), conference registration discounts or waivers (64%) and then funding and recognition opportunities through travel awards, research grants, prizes, etc. (58%). Furthermore, around a third (38%) of the societies offered discounts or waivers of APC in society-affiliated journals. Other benefits included a broad variety of items ranging from free newsletters, discounts on purchasing books from supporting publishers, discounts on joining partner societies, access to society’s physical library, podcast series, field trips, job placements and even the use of a designated suffix after a member’s name. Most societies (67%) offered at least three types of membership benefits in many different combinations (figure 5).

## 5. Discussion

Our survey revealed the distribution of current practices related to structuring individual membership fees, benefits and characteristics of learned societies in ecology and evolution. We discuss our findings, acknowledge limitations and then provide recommendations for making membership fees more transparent and inclusive.

### (a) Individual regular membership, student, and postdoctoral researcher fees

We found that regular individual membership fees often exceed 50 USD per year per member (57% of the surveyed societies). The membership fees were usually (83% of the surveyed societies) discounted for students to around 50% of the regular fee. However, only 26% of the societies offered similar concessions to other early-career researchers after PhD completion, and they were sometimes only eligible for up to 5 years. There are three major points to consider for interpretations and implications of our findings.

First, we should not see each membership fee as a one-off expense or separately from other memberships. According to two large-scale cross-disciplinary surveys conducted by Roscoe [16], 41−44% of respondents were members of at least three societies and senior researchers were more likely to join multiple societies than junior researchers. Survey respondents also identified the loss of a funding source as the important barrier to joining a society and the most common reason behind letting their membership lapse. Such a pattern could be driven by the limited affordability of memberships to junior researchers who, despite concessions, cannot afford to pay for multiple memberships or for consecutive years, especially if their financial situation changes. Similarly, an ad hoc survey among the 21 authors of this work revealed that we held, on average, 3.7 memberships in 2023 (median = 3, min = 0, max = 11), but had to pay out of pocket for, on average, 1.7 memberships (median = 1, min = 0, max = 11). Four of the authors were eligible for free memberships, thus lowering the proportion of the fees that had to be paid privately. One author noted that their employers would not cover any membership fees and they had no other option but to pay out of pocket. Over half (13 out of 21) wished they could become a member of additional societies in 2023 but could not afford to (electronic supplementary material, table S3).

Second, we can see that three-quarters of the societies surveyed may assume that concessions are no longer needed after PhD completion. Such an assumption could be interpreted as a legacy of the times when career prospects and financial realities were more optimistic for early- and mid-career researchers. As of the twenty-first century, survey after survey shows that postdoctoral researchers face deteriorating career prospects linked to growing job and financial insecurity, competitiveness and earnings disproportionate to the increasing workloads [17–20]. Many have to carry student debts that are also growing in recent years for PhD holders, and which tend to disproportionately affect minority groups [21]. Furthermore, the economically precarious postdoctoral stage is getting longer and now it often takes over 10 years to reach relative stability and benefits of a permanent role, if ever reached [22–24]. Furthermore, mid-career is often the period of personal lives in which many face the financial implications of starting a family or caring for dependents [25]. Thus, the postdoctoral stage is when the most vulnerable members of the academic community slow down their careers or leave academia altogether [23].

Third, structuring fees by career stage ignores individual variation in access to resources, including research funding as well as personal funds. From the perspective of researchers or students from well-funded laboratories and organizations, the current fee amounts may seem reasonable and concessions generous. However, funding in many countries has shifted from internal or institutional to increasingly competitive, external and/or project-based funds [26,27], which tend to be disproportionately concentrated in the hands of elite researchers and institutions [27–29]. At the same time, research funders may not allow the use of research grants for professional membership fees (e.g. Australian Research Council Discovery Project grants cannot be used for membership payments) or researchers may be limited by internal institutional policies (e.g. University of New South Wales normally allows one membership payment per researcher to be paid from grants, and CNRS none, while others would not allow paying for Masters students). Thus, we need to also be able to empathize with the situation of many who find it difficult to pay for professional belonging, especially where they are assumed to have financial resources because they have already completed their PhD program or live in a relatively wealthy country.

### (b) Country-level fee discounts and waivers

Our survey revealed that most international societies relevant to ecology and evolutionary biology are located in the Global North countries (especially the USA and UK) and societies in the Global North charge higher fees compared to societies based in the Global South (or in Africa, South America and Asia). Around half of the societies surveyed have discounted or waived fees based on the country of residence of the members. Country of residence is occasionally used to impose increased fees for overseas members, especially within societies based in the Global South. This raises three important questions for readers to ponder.

First, why does country-level fee differentiation exist? Concessions based on country of residence are appealing and popular because it is easy to verify eligibility by looking at members’ institutional affiliation. Country-level concessions may appear equitable because categories of countries based on their economic development generally overlap with total research funding per country [30]. What is seldom noted, however, is that country-level discounts usually only apply to researchers from least developed economies (Global South); thus, countries that are in the middle of the development/wealth spectrum are often bundled together with top-income countries, which seems far from equitable.

Second, do discounts according to country classification (Global North versus Global South or development index) function as proposed in providing equitable access to learned societies? Observational studies can shed some light on this question. Particularly, the two cross-disciplinary global surveys by Roscoe [16] found that early-career researchers from Asia or Africa were less likely to be society members than those from North America or Europe. This observation may be a sign that society membership fee discounts are insufficient, and that the reduced fees are still prohibitive to potential members from many Global South countries. However, there could be other drivers of the observed membership patterns, such as junior scientists are not aware of the existence of the learned societies, they are not encouraged to join international societies by their mentors, or the membership benefits cannot be realized for the members from the Global South.

Third, is the country of residence a good proxy for the ability to pay membership fees? As discussed earlier, a simplistic country-level fee discount (or increase) ignores often vast within-country heterogeneity in personal wealth, income and research funding. It also fails to capture other dimensions of diversity or circumstances beyond the current country of affiliation, for example, being a recent immigrant from a developing economy, having part-time or no employment, or being a member of groups minoritized in academia, such as minoritized racial, ethnic and gender identities. Thus, societies introducing other types of concessions that are based on personal characteristics or considering fee discounts/waivers based on individual circumstances may provide more equitable alternatives to current discount policies.

### (c) Individual-level discounts and waivers

In our survey, we found evidence of concessions being offered for all of the following member types: students, postdoctoral researchers, retired/emeritus, unemployed, employed part-time, junior (pre-university), family, educators/outreach/communication non-academic specialists, general community/public and other categories. However, there are societies that offer no discounts (9%) or only have discounts for students (21%). Most of the time (61%) we found only one or two concession categories from our list, which were typically for students, postdoctoral researchers or retired members. Nevertheless, we also identified 15% of societies with complete or partial fee waivers on individual request, but fewer with no payment or discretionary payment options (6% in total). We provide three important considerations when thinking of these findings.

First, student, postdoctoral researcher and emeritus categories could be considered ‘traditional’ concessions, based on an assumption of a linear, uninterrupted and ascending, academic career path [31]. Not surprisingly, these three concession categories were the most common in our survey; other types of concessions are still uncommon. We argue that consideration of other concessions is critical, as they normalize and accommodate both deviations from the traditional career trajectory and what member categories are considered ‘acceptable’ by learned societies. Societies that are more open and supportive to junior (pre-university) members, non-academics, families or people on limited or with no employment are the ones truly embracing the spirit of EDI and open science [32].

Second, fees proportional to an individual’s annual income are rare. The ‘sliding scale’ approach has been historically used to provide more equitable access to medical services [33] and it could in principle work for any income level. To be effective, the scale has to capture a globally relevant range of incomes rather than be based on typical academic salaries from developed economies. The fees from the top of the scale have to be balanced to compensate for the lower, or zero, fees at the bottom of the scale. While concessions proportional to income may address inequalities linked to current personal income (including underpayment, part-time work or lack of employment, which may be linked to minoritized racial, ethnic, and gender identities), they cannot deal with inequalities in research funding or past inequities.

Third, complete or partial fee waivers on individual requests may sound like a perfect solution. However, we noticed such waivers may be offered only to a limited range of members and for a limited time. Furthermore, having to prepare and submit a written application for such a waiver creates additional burden and stigma. Stigma may come from having to reveal personal or work circumstances, or discomfort of being subject to the power of a stranger deciding whether one deserves a waiver [34]. Furthermore, ethical concerns arise if we consider that such power imbalances may align with historical lines of division between countries, race, gender or class. To counter this, fee waivers need to be completed and considered without any questions asked—in our survey, we found some examples of such practices. Specifically, out of 169 societies, seven offered a ‘zero fee’ membership option and another three allowed discretionary fee amounts. This number is greater if we consider the additional four societies that offer free membership to everyone. Free memberships could be subsidized through membership fees from well-resourced members, donations (which many societies solicit anyway), or other sources of revenue, as available.

### (d) Societies’ characteristics

Our survey shows that publicly stated commitment to EDI aligns well with having dedicated EDI structures and with more inclusive membership fee structures. The fee structures with more concession categories catered for a greater variety of potential members. EDI-committed societies were more likely to offer discounts based on country of residence or fee waivers on request. While this all sounds like reasons to rejoice, there are also three missing pieces here.

First, it might be easily overlooked that around half of the societies captured in our survey did not have public EDI statements and/or dedicated EDI structures. These statements and structures are needed to drive the development of effective policies and actions directed at bringing and supporting diverse members. Lack of diversity has plagued learned societies since their origin and progress towards greater EDI is frustratingly slow [35–37]. This is perhaps reflected in our findings related to poor consideration of equity of the membership fee structures, overall.

Second, societies with public EDI structures were more likely to offer traditional fee waivers for students, postdoctoral researchers and retired members, fee waivers on individual requests, but not other types of flexible or ‘no questions asked’ discounts accommodating personal circumstances. This may be explained by the overall low frequency of the latter types of concessions in the dataset. Implementing such trust-based concessions could be seen as risky by learned societies, but is not science largely based on building trust [38–40]?

Third, it is unclear how the EDI statements, structures and fees are mechanistically linked to each other. Specifically, do policies and structures advocate for more inclusive fee structures and remove obstacles to a diverse membership base? Or does a diverse membership base push societies towards adapting mission statements and creating support structures? What is the most effective path towards transforming culture and climate and providing equitable access for all? Perhaps we need all of it happening at once [41–43].

### (e) Membership benefits

The tangible benefits of individual society memberships are hard to capture because of their diversity. Our survey categorized information provided on the webpages into six broadly defined benefit categories. We showed that the benefits offered by the majority of the societies typically represent three of these categories. These most commonly are: free or discounted journal subscriptions, conference registration discounts or waivers, and funding and recognition opportunities through travel awards, research grants and prizes. All of these can be considered as substantial, or even critical, for career progression, but especially for groups and individuals that cannot afford to pay membership fees [5].

## 6. Recommendations

Finally, we offer eight actionable recommendations to make membership fees of learned societies in ecology and evolution more transparent and equitable. We believe that institutional transparency and equity are needed to ensure that learned societies are inclusive and diverse, representing and supporting all stakeholders who would benefit from society memberships.

1. Raise awareness about EDI among the society members and the leadership. Buy-in from leadership and/or those with the privilege and power to create change will be essential for changing the membership fee structure.
2. Collect comprehensive membership diversity data, including the intersection of personal characteristics and ability to pay membership fees. Using such data, identify areas that are deficient or require improvement, consider implementing more inclusive practices and evaluate changes when new fee structures or EDI initiatives are introduced.
3. Make the diversity of the past and current membership base and the leadership team publicly visible, and consider intersectional aspects of diversity, e.g. by annually publishing aggregated data summaries. Making the invisible visible is key to driving action towards greater institutional equity.
4. Survey society members and relevant non-members, including lapsed members, on their fee structure preferences, and collect feedback after implementing changes. Pay special attention to the voices of historically underrepresented and marginalized groups (this includes minoritized racial, ethnic, class and gender identities) and consider sliding-scale, discretionary and zero-fee membership options, even if they require an honour system and are based on trusting the members.
5. Consider actively broadening a membership base to non-traditional contributors from outside academia and make it affordable for them. Recognizing the value of more diverse individuals, welcoming them and providing tailored access can benefit academics and scientific research as a whole.
6. Make generous concessions for postdoctoral researchers. They are a large group often treated as an invisible part of the academic workforce, and increasingly burdened by a precarious economic situation.
7. Remove time limits for all concessions. Concessions are needed as long as a person is affected by their professional or personal circumstances and it is not equitable to assume that their situation will change dramatically after a year or a few years.
8. Be clear about membership benefits and how they apply to different member groups. Have them explicitly listed on the membership page, regularly updated, and linked to other relevant documents, as applicable.

## 7. Limitations

The results of our survey should be considered in light of four limitations. First, the survey only presents a snapshot of data at a given point in time. Thus, no inferences of time trends can be drawn. Second, we had no information on the membership base composition of the surveyed societies. Thus, we could not relate how this aspect is linked to membership fee structures. Third, we did not extract the full scale of available membership options available at some societies because we focused on the most common and comparable broader membership categories. Fourth, we excluded the ‘lifetime membership’ category from our data collection because we assumed that this option is not viable for people with limited or precarious financial resources.

## 8. Conclusions

Current membership fee structures often do not take into account the realities of diverse individual members. By creating barriers to professional belonging and membership benefits, societies themselves may contribute to research career precarity and inequality and limit the progress of science more generally. On a more positive note, we observed a noticeable alignment between societies with EDI statements and structures and the diversification of their membership options. This brings hope that the ongoing movement towards greater recognition of EDI as a critical aspect of a healthy scientific system will reshape learned societies as a place of opportunities and belonging for all.

## Data Availability

The dataset and analytical code are publicly available on GitHub [44] and archived on the Zenodo Digital Repository [45]. Supplementary material is available online [46].
